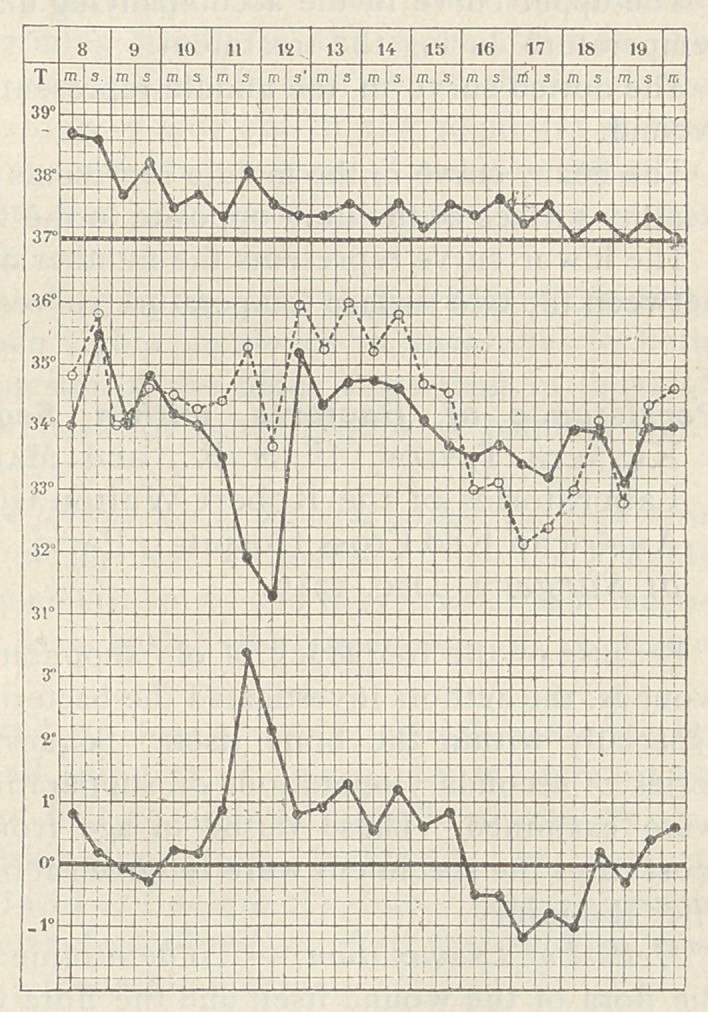# The Moment for Delayed Primary Suture as Indicated by Local Temperatures

**Published:** 1918-03

**Authors:** 


***The Moment for Delayed Primary Suture as Indicated by Local Temperatures.*** By H. VIGNES of G. S. O. S. of Bouleuse. Trans. and Abs. from the *Journal des Praticiens,* March 30, 1918.

Delayed primary suture, usually performed during the first four days after the initial operation, but which may sometimes be performed as late as the tenth or twelfth day, may be carried out by means of stitches inserted with the first operation. Thus anesthesia and surgical treatment need not be repeated. The author, after reviewing the arguments for delayed primary suture, sets forth indications of the time for closing a wound by this method. One must be guided principally by the temperature, the pulse, the pain, and the appearance of the wound.

The writer outlines a case in which a study of the temperature of the wound region compared with the temperature of a relatively healthy portion of the limb determined the time for tying the stitches. A projectile passing through the arm had made a small opening on the exterior face of the limb and had barely perforated the interior face. The important nerves and vessels were so menaced as to render thorough surgical cleansing difficult. The bone was curetted slightly, iodine applied, stitches placed but not tied, and the wound left with a capillary drain.

The temperature of the wound region was recorded by two curves, one (dotted) representing the temperature near the chief opening of the wound, and the other (heavy) representing the temperature on the opposite healthy face of the limb, also under a bandage. On the fourth day after the operation the curves began to diverge, indicating, the writer believed, a condition unfavorable to suture. The lips of the wound were separated and half a tablespoonful of perfringens pus was removed. On the ninth day the curves came together again, indicating, the writer believed, that the perfringens infection had run its course. The wound was in good condition, and four of the stitches were tied. Two days later the remaining stitches were tied. Healing proceeded normally.

The upper curve in the accompanying diagram represents rectal temperature during the treatment.

The dotted curve in the middle represents temperature near the wound.

The heavy curve in the middle represents the temperature on the healthy surface of the limb opposite to the wounded surface.

The lower curve represents the number of degrees of difference between the two surface temperature curves.

## Figures and Tables

**Figure f1:**